# Step by step analysis on gene datasets of growth phases in hematopoietic stem cells

**DOI:** 10.1016/j.bbrep.2024.101737

**Published:** 2024-05-30

**Authors:** Mohammad Elahimanesh, Nafiseh Shokri, Payam Mohammadi, Najmeh Parvaz, Mohammad Najafi

**Affiliations:** a Clinical Biochemistry Department, Faculty of Medical Sciences, Iran University of Medical Sciences, Tehran, Iran; b Clinical Biochemistry Department, Faculty of Medicine, Shahid Beheshti University of Medical Sciences, Tehran, Iran; c Cellular and Molecular Research Center, Faculty of Medical Sciences, Iran University of Medical Sciences, Tehran, Iran

**Keywords:** Hematopoietic stem cell, Signaling pathways, Gene ontology, HSC phases, Microarray

## Abstract

**Background:**

Umbilical cord blood hematopoietic stem cells (UCB–HSCs) have important roles in the treatment of illnesses based on their self-renewal and potency characteristics. Knowing the gene profiles and signaling pathways involved in each step of the cell cycle could improve the therapeutic approaches of HSCs. The aim of this study was to predict the gene profiles and signaling pathways involved in the G0, G1, and differentiation stages of HSCs.

**Methods:**

Interventional (n = 8) and non-interventional (n = 3) datasets were obtained from the Gene Expression Omnibus (GEO) database, and were crossed and analyzed to determine the high- and low-express genes related to each of the G0, G1, and differentiation stages of HSCs. Then, the scores of STRING were annotated to the gene data. The gene networks were constructed using Cytoscape software, and enriched with the KEGG and GO databases.

**Results:**

The high- and low-express genes were determined due to inter and intra intersections of the interventional and non-interventional data. The non-interventional data were applied to construct the gene networks (n = 6) with the nodes improved using the interventional data. Several important signaling pathways were suggested in each of the G0, G1, and differentiation stages.

**Conclusion:**

The data revealed that the different signaling pathways are activated in each of the G0, G1, and differentiation stages so that their genes may be targeted to improve the HSC therapy.

## Highlights

• The high- and down-express genes were identified in each of the G0 and G1 phases and differentiation.
• The genes were visualized on networks.
• The signaling pathways were found in each of the G0 and G1 phases and differentiation.

## Graphical abstract

**Figure:**
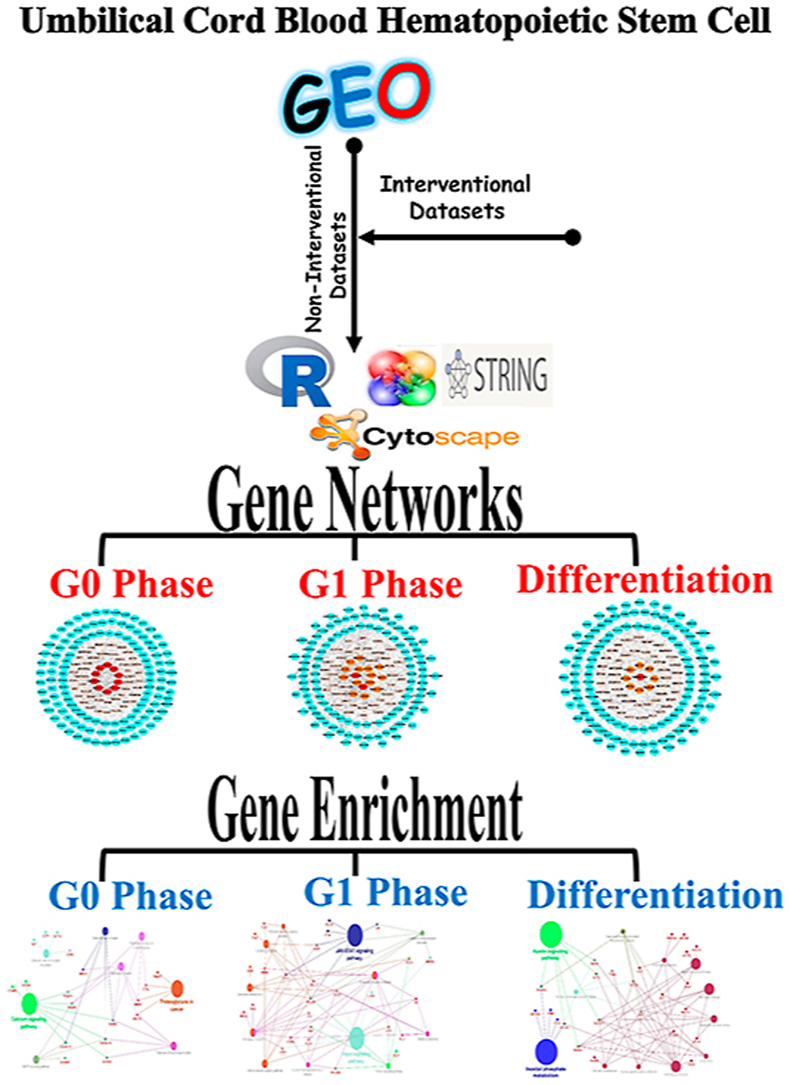

## 1 Introduction

The self-renewal and differentiation into various cells are known as the two fundamental properties of hematopoietic stem cells (HSCs). These features of hematopoietic stem cells have led to their applications in the treatment of blood cell-related malignant illnesses [1]. The hematopoietic stem cells are often derived from bone marrow, peripheral blood, and umbilical cord blood (UCB). The umbilical cord blood is one of the main sources of hematopoietic stem cells, so it is considered a non-invasive and disposal source [2,3]. It is well known that hematopoietic stem cells go through the cell cycle by changing chromatin structure and signaling pathways [4]. Thus, knowing the molecular factors and signaling pathways that can determine the fate of HSCs through the cell cycle and differentiation stages is important for their therapeutic applications.

Some studies reported that the MAPK, WNT, and Notch signaling pathways regulate the proliferation and differentiation of hematopoietic stem cells as well as the ultimate fate of these cells [[5], [6], [7], [8]]. In this regard, some genes such as Hoxb4 and Bmi-1 have been reported to the expansion and proliferation of hematopoietic stem cells [9,10]. C-Myc also increased the expansion of hematopoietic stem cells via p21 and p53 [11]. Using gene expression profiles obtained from microarray and RNA-seq analyses, some studies discovered differently expressed genes and important signaling pathways that played critical roles in the growth, proliferation, and differentiation of HSCs. Some of these studies reported that cyclin E and RB could act as important mediators of cell cycle [12,13]. Thus, knowing and controlling the genes and signaling pathways involved in each stage of the cell cycle of hematopoietic stem cells could improve their clinical applications and approaches.

In the current study, to identify the signaling pathways involved in the cell cycle of hematopoietic stem cells, interventional and non-interventional GEO datasets were crossed and analyzed to find the gene networks in the G0, G1, and differentiation stages. Then, the low- and high-express genes on the networks were enriched to predict the involved cellular pathways in the cell cycle phases. The results of this study might suggest the gene targets in each of the G0, G1, and differentiation stages of HSCs.

## 2 Materials and methods

### 2.1 GEO datasets

We searched the RNA-Seq and microarray datasets related to the transcriptomic data, and selected the GEO databases containing the gene expression data in G0, G1 phases and the differentiation of umbilical cord hematopoietic stem cells (HSCs). 11 datasets were found based on the evaluation of gene expression levels in cell cycle phases, data availability, and text mining of interventional factors (Fig. 1). Among the 11 datasets, the non-interventional datasets (n = 3) were related to the gene profiles in the G0 and G1 phases and differentiation of hematopoietic stem cells, and the interventional datasets (n = 8) were related to the interventional studies that were claimed to affect the growth and proliferation of hematopoietic stem cells. Some characteristics of the chosen datasets are provided in Table 1.

**Fig. 1:**
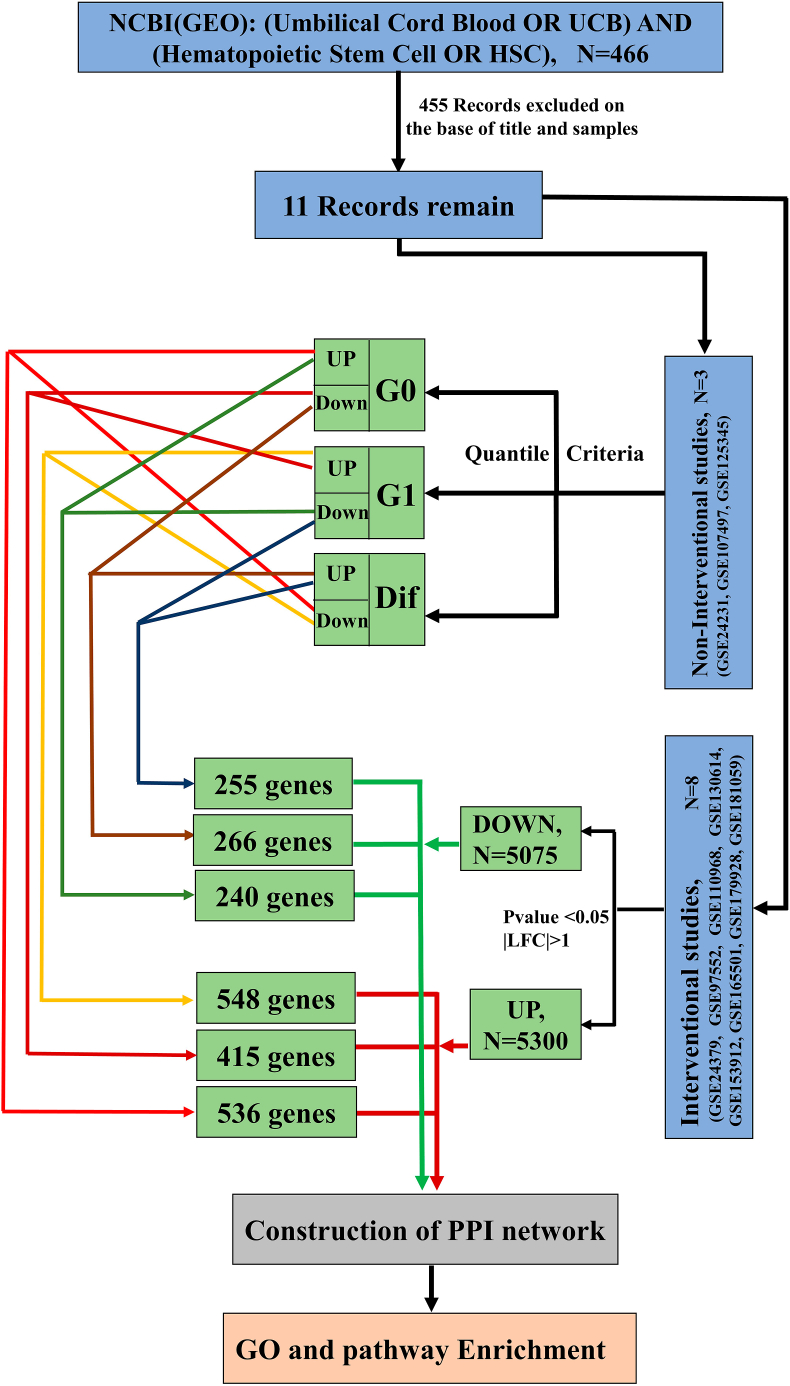
Current research flowchart. Step 1. Data identification and filtration (blue boxes); 466 datasets were found after searching keywords (Umbilical Cord Blood OR UCB) AND (Hematopoietic Stem Cell OR HSC) in GEO database, and finally 11 datasets were filtered by considering the samples of datasets. 8 datasets were related to the studies that investigated the effects of external factors on the growth and proliferation of HSCs, and 3 datasets were related to the studies that determined the gene expression profile of the HSCs in the G0, G1 and differentiation stages. **Step 2. Data processing and analysis (green boxes);** In the non-interventional datasets, the samples related to each of the stages of G0, G1, differentiation were selected according to the quartile criteria. The genes whose expression levels were lower than the first quartile and the genes whose expression levels were higher than the third quartile were determined as down and up genes, respectively. Then, the gene data were shared the up and down genes of the G0 and G1 and differentiation stages with each other to determine which genes change from one cell cycle stage to the next (A, G0 _up_**∩** Dif _down_; B, G0 _down_**∩** G1 _up_; F, G1 _up_**∩** Dif _down_; C, G0 _up_**∩** G1 _down_; D, G0 _down_**∩** Dif _up_; E, G1 _down_**∩** Dif _up_). The differentially expressed genes in each interventional dataset were determined by the criterion |Log_2_FC| > 1 and p-value <0.05. Then, the inter intersections of the up and down genes of interventional studies carried out with the gene groups determined in the non-interventional datasets. **Step 3. Gene network analysis (gray box);** The up and down genes of the G0, G1 and differentiated stages supported with the intervention datasets were used to construct the gene networks. **Step 4. Enrichment (orange box);** The enrichment analysis was performed on the gene network to find the pathways and cellular processes. Dif: Differentiation.

**Table 1:** Datasets used in the study.

GEO	Samples (n)	PMID	Experiment type	Platform	Report (Year)	Type of study
GSE24231	18	N/A	Expression profiling by array	GPL4133	2010	Non-Intervention
GSE107497	19	29701016	Expression profiling by array	GPL17586	2018	Non-Intervention
GSE125345	15	31631013	Expression profiling by high throughput sequencing	GPL16791	2019	Non-Intervention
GSE97552	4	N/A	Expression profiling by array	GPL23270	2020	Intervention
GSE110968	15	30348672	Expression profiling by high throughput sequencing	GPL16791	2018	Intervention
GSE130614	18	31919081	Expression profiling by high throughput sequencing	GPL18573	2020	Intervention
GSE153912	6	N/A	Expression profiling by high throughput sequencing	GPL16791	2021	Intervention
GSE165501	6	35427423	Expression profiling by high throughput sequencing	GPL11154	2022	Intervention
GSE181059	10	35490198	Expression profiling by high throughput sequencing	GPL24676	2022	Intervention
GSE179928	8	N/A	Expression profiling by high throughput sequencing	GPL18573	2021	Intervention
GSE24379	12	22298821	Expression profiling by array	GPL4803	2010	Intervention

### 2.2 Differential expression genes (DEGs)

The non-interventional datasets (GSE24231, GSE107497, and GSE125345) contained the gene sets related to cycle cell phases and differentiated cell lines of hematopoietic stem cells, including MLP (Multipotent lymphoid progenitors), EPC (Endothelial progenitor cells), MPC (Multiple progenitor cell), GMP (Granulocyte-monocyte precursor), and CMP (Common myeloid progenitor), so that their expression data were normalized and annotated using log2 in DESeq2, LIMMA, and Affy packages in RStudio software [[14], [15], [16]]. Then, the low and high expression genes were determined in the G0 and G1 phases and differentiation based on the first (Q1) and third quartiles (Q3), respectively.

Regarding the interventional datasets (GSE97552, GSE110968, GSE130614, GSE153912, GSE165501, GSE181059, GSE179928, and GSE24379), that claimed to change the growth and proliferation by some stimulating factors (such as cytokines, proteins, and culture contents) in HSCs (Table 1), were separately analyzed in RStudio software. The DESeq2 and LIMMA and Affy packages were used to determine the high (LFC >1, p value < 0.05) and low (LFC < −1, p value < 0.05) expression gene sets.

#### 2.2.1 High- and low-express genes in the G0, G1, and differentiation stages of HSCs

To investigate the gene expression changes during the cell cycle phases and differentiation (Dif), the intra intersections (**A**, G0 _up_ ∩ Dif _down_; **B**, G0 _down_ ∩ G1_up_; **C**, G0 _up_ ∩ G1 _down_; **D**, G0 _down_ ∩ Dif _up_; **E**, G1 _down_ ∩ Dif _up_, **F**, G1_up_ ∩ Dif _down_) were identified between the low- and high-express gene sets obtained from the non-interventional datasets so that it could determine differentially the genes involved in the G0, G1 and differentiation stages.

### 2.3 Construction of gene network

The gene networks were created for the gene groups (A, B, C, D, E, and F) using the STRING server (score >0.5, Cluster >3 nodes) [17] and visualized using Cytoscape software (version 3.9.1). Then, the inter intersections were determined between high- and low-expression gene sets (up and down) obtained from interventional datasets and the intra-intersectional gene groups (A, B, C, D, E, and F) on the gene networks.

### 2.4 Gene enrichment

The gene network enrichments were carried out by Cytoscape ClueGO plugins and Shiny GO database (http://bioinformatics.sdstate.edu/go/) [18,19]. The gene networks (n = 6) were independently enriched using KEGG and Gene Ontology (GO).

## 3 Results

### 3.1 The inter and intra intersections were predicted the high- and low-express genes

The high (above third quartile)- and low (under first quartile)-express gene sets (Supplement 1) in each of the G0 (n = 6476 and n = 6466, respectively), G1 (n = 4315, n = 3763, respectively) and differentiation (n = 5911, n = 6243, respectively) stages of hematopoietic stem cells were determined from the gene samples (GSM) of non-interventional datasets (GSE24231, GSE107497, and GSE125345) (Table 2). Furthermore, the data were supported with high (LFC > +1, P-value <0.05) -and low (LFC < −1, P-value <0.05) -express gene sets (Supplement 2) obtained from the intervention datasets (GSE97552, GSE110968, GSE130614, GSE153912, GSE165501, GSE181059, GSE179928, and GSE24379) (Table 3).

**Table 2:** Samples related to G0, G1 and differentiation stages.

Cell Phase	GSE24231	GSE107497	GSE125345	Sample (n)	UP Genes (n)	DOWN Genes (n)
G0	GSM595960 GSM595967 GSM595969	GSM2869312 GSM2869313 GSM2869314	GSM3569313 GSM3569314 GSM3569315	9	6476	6466
G1	GSM595961 GSM595968 GSM595970			3	4315	3763
Differentiation		GSM2869315 – GSM2869330 (16 samples)	GSM3569319 – GSM3569327 (9 samples)	25	5911	6243

**Table 3:** Differential express genes in intervention datasets.

	GSE24379	GSE97552	GSE110968	GSE130614	GSE153912	GSE165501	GSE179928	GSE181059	Total unique genes^a^
UP Genes (n)	409	40	828	291	167	2994	3815	53	5300
DOWN Genes (n)	683	2	933	97	109	3788	4381	188	5075

a , Unique genes were estimated on the unions of Up and Down genes of datasets.

The up- and down-gene expression levels were reported to change during cellular phases in non-interventional datasets. The intra intersection results showed that some highly expressed genes in the differentiation stage (Dif _up_) of hematopoietic stem cells had low expression levels in the G1 (G1 _down_) and G0 (G0_down_) phases (n = 255 and n = 266, respectively). Furthermore, the lowly expressed genes in the differentiation phase (Dif _down_) of hematopoietic stem cells had high expression levels in G1 (G1_up_) and G0 (G0_up_) phases (n = 548 and n = 536, respectively). The low-express genes in G0 phase (G0_down_) but the high-express in G1 phase (G1_up_) were 415 (Supplement 3). The inter intersection results between the up genes of interventional datasets (n = 5300) and three groups (F (G1_up_ ∩ Dif _down_), A (G0_up_ ∩ Dif _down_), B (G0_down_ ∩ G1_up_)) that had the higher gene expression levels in the G0 and G1 as compared to the differentiation in non-interventional datasets were confirmed the roles of some genes in the G0 and G1 phases. Furthermore, the inter intersections between the down genes of the interventional datasets (n = 5075) and the genes that had high expression levels in the differentiation and G0 in non-interventional datasets (E (G1 _down_ ∩ Dif _up_), D (G0_down_ ∩ Dif _up_), C (G0_up_ ∩ G1_down_)) were identified (Fig. 2, Supplement 4).

**Fig. 2:**
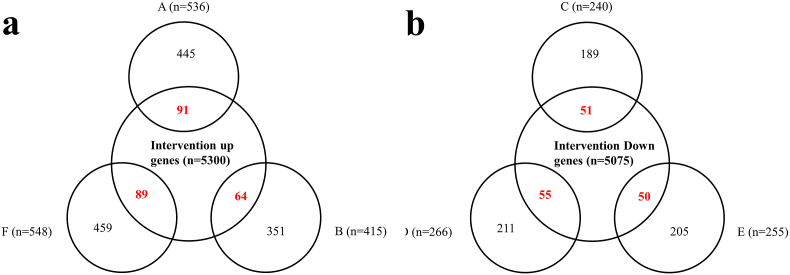
Venn diagrams of dataset inter and intra intersections. (**a**) The common genes between up genes (n = 5300) of intervention datasets and the F (G1_up_ ∩ Dif _down_ (n = 548)), A (G0_up_ ∩ Dif _down_ (n = 536)) and B (G0_down_ ∩ G1_up_ (n = 415)) groups. (**b**) The common genes between the down genes (n = 5075) of the intervention datasets and the E (G1 _down_ ∩ Dif _up_ (n = 255)), D (G0_down_ ∩ Dif _up_ (n = 266)), and C (G0_up_ ∩ G1_down_ (n = 240)) groups.

### 3.2 Gene networks were constructed on the intersections of gene groups

The inter intersections between the non-interventional gene groups (blue nods) and the interventional data (nods at light pink to red ranges) were applied to construct six gene networks (Fig. 3A–F). The hub genes were determined based on network centrality parameters (Table 4).

**Fig. 3:**
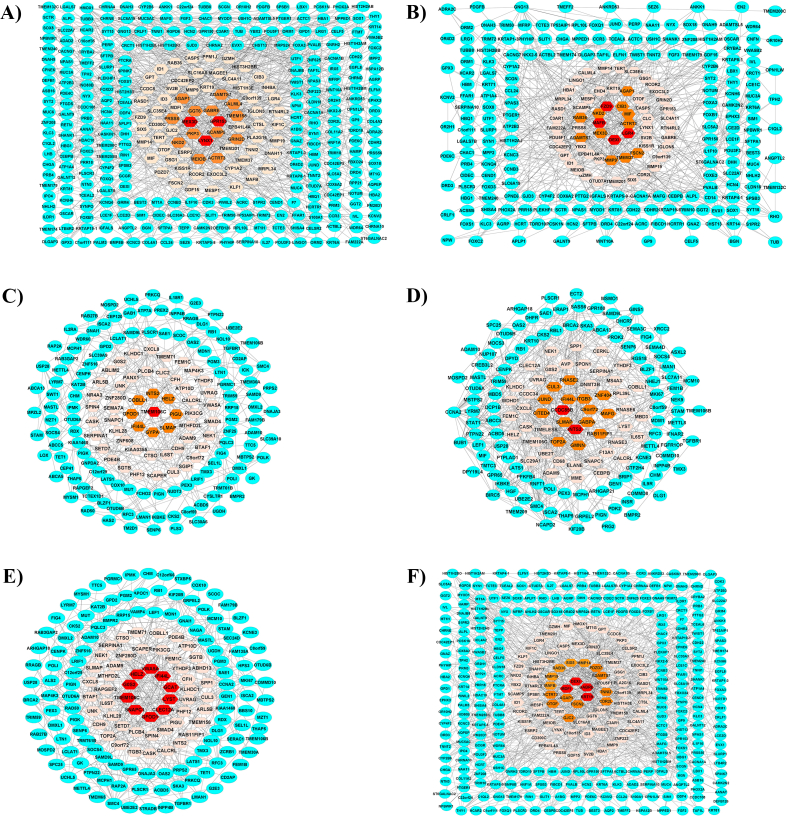
Gene networks. The gene networks were created for the gene groups (A (G0_up_ ∩ Dif _down_), B (G0_down_ ∩ G1_up_), C (G0_up_ ∩ G1_down_), D (G0_down_ ∩ Dif _up_), E (G1 _down_ ∩ Dif _up_) and F (G1_up_ ∩ Dif _down_)). Each network includes two kinds of nodes, indicated as the blue circular nods () in the peripheral section and red (High score) to light pink (Lower score) hexagonal nods () in the central section of the network. The blue nods corresponded to the genes obtained from non-intervention datasets, and the red to light pink nods corresponded to the genes that were confirmed by intervention datasets.

**Table 4:** The gene networks.

Networks	Node^a^, Edge	STRING networks Pvalue**	Hub genes
A group (G0_up_**∩** Dif_down_)	307,716	10^–18^	MMP9, KRT19, PVALB, GPT, TERT, GNG13, PACSIN1, MYOD1
B group (G0_down_**∩** G1_up_)	260,541	10^–16^	MMP9, POU5F1, KRT19, ATP2B3, TERT, NKX2-5, GNG13
C group (G0_up_**∩** G1_down_)	174,379	10^–7^	TOP2A, PIK3CG, CCNA2, SMC4, CXCL8, SMAD4, RFC3, CUL3
D group (G0_down_**∩** Dif_up_)	164,469	10^–8^	TOP2A, CCNA2, BUB1, RFC3, SMC4, WDHD1, BIRC5, MCM10, GMNN, CXCL8
E group (G1_down_**∩** Dif_up_)	173,287	10^–9^	CXCL8, PIK3CG, SMAD4, CUL3, SMC4, SGIP1, CASK, BTAF1
F group (G1_up_**∩** Dif_down_)	360,895	10^–14^	MMP9, POU5F1, KRT19, GPT, TERT, HMOX1, MYOD1

a , Only the nodes connected by the high-score edges in the STRING. **, PPI enrichment P-value.

### 3.3 The signaling pathways were predicted by enrichment of gene networks

Fig. 4 shows the most important signaling pathways in each network. The signaling pathways regulating pluripotency of stem cells (p 7.00e-09), WNT (p 1.12e-08), Ras (p 8.74e-10), MAPK (p 2.44e-07) and pathways in cancer (p 4.01e-06) suggested to be involved in the G1 phase. Also, the Inositol phosphate metabolism (p 6. e−06), Apelin signaling pathway (p 3.4e-8), Calcium signaling pathway (p 1.5e-05) and Proteoglycans in cancer (p 2.4e-03) suggested to be related to the G0 phase. Furthermore, NOD-like receptor signaling pathway (p 7.67e-06), Hippo signaling pathway (p 3.7e-05), Th17 cell differentiation (p 9.00e-07), and Jack-Stat signaling pathway (p 3.99e-08) proposed to be involved in the differentiation stage.

**Fig. 4:**
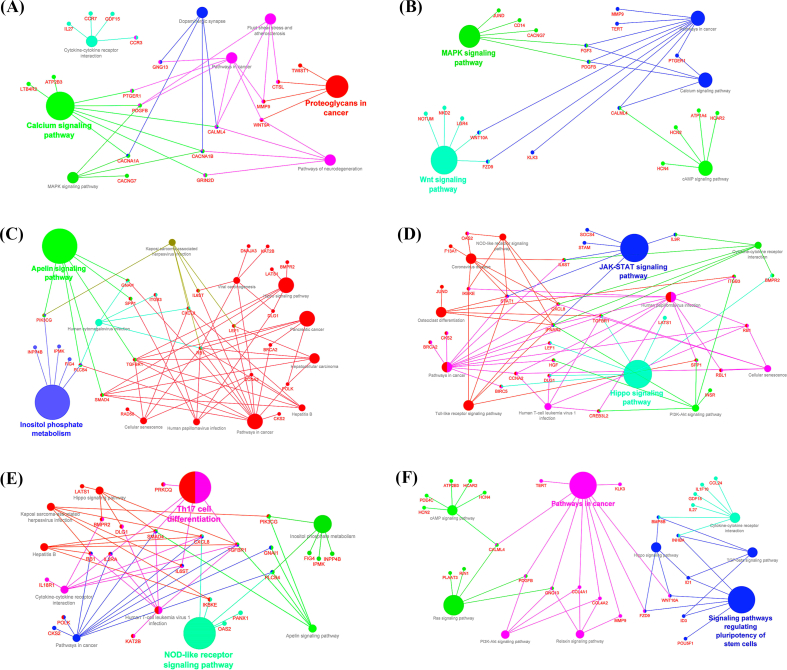
Enrichment of the gene networks. The gene networks ((A (G0up ∩ Dif down), B (G0down ∩ G1up), C (G0up ∩ G1down), D (G0down ∩ Dif up), E (G1 down ∩ Dif up) and F (G1up ∩ Dif down)) were enriched using the KEGG pathways. In each network, the genes are shown in the red color and the cellular signaling pathways are shown in the different colors. Moreover, the node size is related to the signaling pathway's importance in the network.

GO function enrichment analysis (Biological process and Molecular function) was carried out on the gene networks (Supplement 4). The biological processes including the cell-cell signaling, epithelium development, cellular protein localization and the molecular functions of DNA binding transcription factor, RNA polymerase *cis*-regulatory, and adenyl ribonucleotide binding are actively suggested in the G0 phase. Also, the biological processes of epithelium development, regulation of ion transport, cell-cell signaling and the molecular functions including channel and transporter, transcription factors bind to DNA are suggested in the G1 phase. Furthermore, the biological processes such as cell cycle, cell cycle regulation, cellular proteins and macromolecule regulation, and the molecular functions of adenyl ribonucleotide binding, and chromatin binding factor are proposed in the differentiation stage (Supplement 5, A-F).

## 4 Discussion

Hematopoietic stem cells (HSCs) can differentiate into different types of blood cells, including MLP, EPC, MPC, and CMP. In addition to ease of preparation and access, lack of risk for donors, high sensitivity for inducing tolerance, low levels of alloantigen and HLA (Human Leukocyte Antigen) surface antigens, and appropriate compatibility are known as the desired characteristics of umbilical cord blood HSCs. Since controlling the cell cycle and differentiation of HSCs could improve the therapeutic goals, thus maintaining HSCs in each of the proliferation and differentiation phases requires knowing the active cellular pathways. Considering that a cell goes through different phases during its life, including the G0 and G1 phases and differentiation, it seems to understand which genes must control signaling pathways. In this study, we tried to predict the active cellular signaling pathways in each of the G0, G1 and differentiation stages. After determining the high- and low-express genes in each of the cell cycle and differentiation stages, some genes were confirmed using the datasets that examined the expression of HSC genes under interventional conditions. Thus, the hub genes were identified on the gene networks constructed from the intra-intersections between the G0, G1, and differentiation gene sets and supported by the interventional gene data. The enrichment of genes in the gene networks identified the important signaling pathways in each of the G0, G1, and differentiation stages. In the following, some genes involved in these pathways are discussed.

The study results showed that Inositol phosphate metabolism, Calcium signaling pathway, Apelin signaling pathway, and Proteoglycans in cancer were suggested to be the most important pathways in the G0 phase of HSCs. It has been shown that inositol phosphate, as a secondary messenger, can increase the concentration of calcium in cells. Thus, the inositol phosphate-calcium signaling pathway is crucial to control some functions, including muscle contraction, cell secretion, metabolism, cellular development and differentiation. Despite the general agreement that IP3 (Inositol triphosphate) plays an important role in intracellular Ca^2+^ mobilization, debates over the precise mechanisms behind inositol phosphate metabolism and Ca^2+^ signaling have arisen [20]. Phosphatidyl inositol may aid cells in maintaining the G0 phase by protecting them from oxidative stress and maintaining their stability [21]. Phospholipase C-β4 (PLCβ4) is responsible for the hydrolysis of phosphatidylinositol 4,5-bisphosphate (PIP2), the inner membrane component, generating the second messengers IP3 and DAG. PLCβ4 has an important role in the metabolism of inositol phosphate so it is known as a regulatory target in the inositol metabolism [22]. INPP4B, IPMK and FIG4 are other genes related to the inositol metabolism pathway that are predicted in our study. Future research can examine this pathway and genes in the G0 phase of hematopoietic stem cells. Moreover, the calcium signaling pathway was proposed to be involved in the G0 and G1 phases. The cell proliferation and maintenance depend on intracellular calcium concentration. P53 affects the expression of OCT4 and Nanog genes and plays a role in arresting the cell cycle so it is suggested as an important obstacle to maintain self-renewal and pluripotency in stem cells. The intracellular calcium regulates the activation of an intracellular protease called calpain, which can break down P53 and prevent its activity. Evidence shows that the amount of this enzyme increases in the G1/S phases of the cell cycle. The intracellular calcium also changes the c-myc gene expression levels, which is a master transcription factor and can increase the expression of some important genes in the self-renewal of stem cells, such as OCT4 and Nanog [23]. When the cells that are on the border of the G0/G1 phase receive signaling messages and appropriate growth factors, the concentration of calcium increases in them, and they can improve the cell cycle. Calcium is needed for many enzymes that play important roles in DNA replication and nucleotide synthesis during the cell cycle [24,25]. It has been shown that the intracellular calcium concentration and calmodulin play significant roles in regulating the cell cycle [26]. A variety of calcium-dependent processes are mediated by voltage-dependent calcium channels (CACNA1A), plasma membrane Ca^2+^ transporting ATPase 3 (ATP2B3), and calcium voltage-gated channel subunit alpha1 B (CACNA1B), which also play a role in mediating the entry of calcium ions into cells [27,28]. Based on the outcomes of our prediction and the functions of the genes that were discovered about the calcium pathway, future research may focus on understanding how intracellular calcium concentration can change cell phases of hematopoietic stem cells through channels that import calcium into the cell or carriers that can bind to the intracellular calcium. Apelin can also interact with the Apelin receptor (APJ) in Apelin signaling pathway. Apelin and APJ are widely expressed in diverse tissues. It is reported that adding the Apelin ligand to the cell medium AGM (Aorta-gonad-mesonephros), HSPCs as well as erythroid cells were created in the next generation [29]. It has also been reported that hematopoietic stem cells regulate the size of blood vessels through the Apelin signaling pathway and Angiopoietin/Tie2 interactions [30]. Furthermore, Apelin via the TGFBR receptor can activate SMAD and reduce Epithelial-mesenchymal transition (EMT) in kidney cells preventing chronic kidney disease (CKD) [31]. GNAI1 G protein subunit alpha i1 is abundantly expressed in immune cells and participates in the G protein-coupled receptor (GPCR) and non-GPCR signaling pathways. GNAI1 and GNAI3 regulate cytokine responses to bacterial infections [32]. SPP1 is also essential to adhere osteoclasts to the mineralized bone matrix probably via vitronectin receptor [33] so that this event may maintain HSCs in G0 phase before the release into the circulation. Since the Apelin pathway is related to inositol metabolism, the pathways in cancer and senescence through central genes, the relationships between these pathways may maintain the hematopoietic stem cells in the G0 phase. Proteoglycans, including Phosphacan, Glypican and Syndecan regulate cell proliferation, and cell volume [34]. V. Iozzo et al. reported that decorin can suppress β-catenin and Myc, and increase the activity of the P21 protein, a cell cycle regulator. They suggested that perlecan via VEGFR2 causes cellular migration and proliferation. They also proposed that proteoglycans may have an impact on intracellular signaling pathways that determine the cell fate [35]. HSCs located in the special microenvironment of bone marrow, under the influence of growth factors, cytokines and other signaling messages, can divide and generate mature blood cells, and be released into the bloodstream. HSCs adhere to the bone marrow niche by varieties of proteoglycans, including decorin, glypican, and other structural supporting molecules such as keratin. The lack of communication between the hematopoietic stem cells and their niche can affect the cell proliferation in the bone marrow [36]. Studies also reported that the effects of glycosaminoglycans such as hyaluronic acid on the cell surface receptor of CD44 stimulate Twist1 expression in cells [37]. The increase of Twist expression in mouse hematopoietic stem cells led to an increase in G0 phase and a decrease in the population of differentiated cells [38]. Cathepsin L, an endolysosomal cysteine protease, increases its activity in peripheral blood mononuclear cells (PBMNCs) and bone marrow mononuclear cells (BMMNCs) of patients with acute myeloid leukemia (AML) [39]. HSCs in the bone marrow regulate their proliferation and quiescence by continuously expressing cathepsin L and its inhibitor, CTLA2 [40]. MMP9 was reported to be involved in the breakdown of extracellular matrix in normal physiological processes such as embryonic development, reproduction, and tissue remodeling. The increased MMP9 correlated to successful HSPC transplantation. MMP9 also plays an essential role in the migration of HSCs from the bone marrow niche into the blood [41,42]. Moreover, MMP9 caused the transfer of hematopoietic stem cells from the quiescence niche to the proliferative niche [43]. Considering the multiplicity of the proteoglycan family in cells and the different roles that they play, such as starting an intracellular signaling pathway and changing the expression of genes, it is possible to investigate more precisely the different proteoglycans that can play a role in determining the growth phase of hematopoietic stem cells.

MAPK, WNT, regulating pluripotency of stem cell signaling pathways and pathways in cancer are highly suggested active in the G1 phase of HSCs in this study. The MAPK signaling pathway plays an important role in the regulation of proliferation in mammalian cells [44]. Furthermore, RAS/MAPK signaling pathway increases cell survival [45]. AP-1 transcription factor subunit (JunD), a nuclear transcription protein from the Jun family, interacts with members of the Fos family and other Jun proteins (c-Jun or JunB) to form the AP-1 transcription complex [46]. JunD relates to the development of mammalian cells during the embryonic period and interacts with EGFR to play a pivotal role in cellular proliferation and cell cycle arrest/senescence [47]. JunD by affecting the P53 protein protects cells against oxidative stress, apoptosis, and senescence [48]. CACNG7 is a voltage-dependent calcium channel that can be activated by neurotransmitters, hormones, and growth factors, and leads to the activation of MAPK signaling pathway via the changes in intracellular calcium [49]. CD14 is a glycoprotein on the plasma membrane of myeloid cells. This receptor can activate the MAPK and NF-ΚB signaling pathways [50]. It seems possible that proteins such as FGF and PDGFB, whose roles in the growth and proliferation of cells have been discussed before and are also predicted in our study, can play a role in the G1 phase in HSCs by stimulating the MAPK pathway. The WNT (Wingless‐related integration) signaling pathway plays an important role in the development of hematopoietic stem cells during the embryonic period and the differentiation of cells into specialized blood cell lines [51,52]. WNT and Notch signaling pathways can increase proliferation and self-renewal in HSCs so, the WNT pathway is necessary for proliferation and initiation of cell cycle, and the Notch pathway prevents HSC differentiation [53]. WNT is a glycoprotein that binds to its receptors, frizzled and LRP, on the surface of cells. Wnt10A increases cellular expansion and self-renewal via activating β-catenin and LEF/TCF [54]. Fzd and LRP transmembrane proteins are involved with the Wnt pathway [55]. Grainger et al. reported that Wnt9a induces β-catenin in hematopoietic progenitor and stem cells via Fzd9b receptor [56]. Furthermore, LGR4 is a GPCR that binds R-spondins and activates the Wnt signaling pathway in the cells [57]. It is also reported that NOUTM and NKD can modify the activity of the WNT pathway [58,59]. The signaling pathways regulating the pluripotency of stem cells preserve the pluripotency features of stem cells [60,61]. INHBA/TGFB1 complex activates SMAD2/3 and improves stem cell characteristics in cancer cells [62]. An inhibitor of DNA Binding (ID1/3) is suggested to be a therapeutic target in hematological malignancy [54]. ID1 prevents LT-HSC transition into ST-HSCs and instructs daughter cells from LT-HSC divisions to remain in this primitive form [63]. ID1/3 is important for the creation of HSPC during the embryonic period and also for maintaining their pluripotency properties [64]. OCT4/POU5F1 complex, along with other transcription factors such as MYC, SOX2 and NANOG can maintain the pluripotency property of stem cells [65]. Huang et al. showed that OCT4 enhances the expansion of hematopoietic stem cells in ex vivo via HOXB4 [66]. Pathways in cancer include a set of signaling pathways that cause uncontrolled cellular proliferation [[67], [68], [69]]. Cross-talking between these pathways can involve many proteins related to the proliferation phase in the cells including HSCs [70,71]. The predicted pathways related to the pluripotency of stem cells also include a set of genes and cell pathways. Our results showed that these signaling pathways and their involved genes have been closely related to each other, and investigating the relationships between these signaling pathways and how they are regulated can be a field of future research in HSCs.

The results suggested the Th17 cell differentiation, NOD-like receptor, JAK-STAT and Hippo signaling pathways are highly active in the differentiation of hematopoietic stem cells in this study. The hippo signaling pathway can control growth and proliferation in stem cells [72,73]. Furthermore, the hippo signaling pathway plays an essential role in the self-renewal and differentiation of hematopoietic stem cells in Xenopus and Drosophila melanogaster [74,75]. The hippo signaling pathway is also involved in the differentiation of human megakaryocytes and the function of mouse hematopoietic stem cells [76]. It has been shown that some proteins, such as LATS1, MTS1/2 and YAP/TAZ in the hippo signaling pathway, can have an important role in the homeostasis of bone marrow HSCs [77]. The YAP1/TAZ complex in the hippo signaling pathway can be activated by LEF1 (lymphoid enhancer binding factor 1), which is a transcription factor involved in the signaling pathway [78]. BMPRs are a group of serine-threonine kinase receptors that, by binding to their ligands (BMPs), play an important role in bone formation during the embryonic period. The hippo signaling pathway is employed by members of the BMP family to control VEGFR2 and Notch signaling pathway [78,79]. TGFBR/TGFB is also cross talked with the hippo signaling pathway to proliferate cardiomyocyte cells [79]. DLG1 is reported to relate to the HSC maintenance, proliferation, and polarity [80]. The hippo signaling pathway increases the expression of BIRC5, which is one of the anti-apoptotic proteins [72]. The hippo pathway plays a role in inflammation and the immune response to viruses and foreign agents through its effect on the expression of some cytokines such as cxcl8, as predicted in our study. The hippo pathway appears to collaborate with the JAK-STAT and pI3K-AKT pathways in differentiated cells derived from HSC. The JAK-STAT signaling pathway is involved in various cellular processes such as growth, hematopoiesis, proliferation, and differentiation. In drosophila, this pathway plays a significant role in the maintenance of HSCs and the formation of lymphoid lineages [81]. Functional hematopoiesis depends on the JAK-STAT pathway. Erythropoietin, thrombopoietin, and other interferons can activate the JAK-STAT pathway and cause the proliferation of HSCs [82]. Some proteins, such as SOCS, STAM, IL6, LIF, and CNTF affect the JAK-STAT signaling pathway [83]. It suggests that future research can focus on analyzing the Jack-STAT pathway and the impact of the change in each gene's expression indicated by our study in this pathway on differentiated HSCs. NOD-like receptor signaling pathway (NLR) may act through MAPK and NF-κB signaling pathways and the inflammasome. The inflammasome in the NOD-like receptor pathway activates caspase 1, which can cleave pro-inflammatory cytokines and causes immune system responses [84]. It has been shown that IKBKE plays an important role in the response of the immune system [85,86]. Other proteins, such as CXCL8, OAS2 and PANX1 are involved in NOD-like receptor signaling pathway [[87], [88], [89]]. Th17 cells are a group of T helper cells that can secrete IL17 and play an important role in the defense of the immune system. IL6 upregulates IL21 and IL23R to differentiate the naive T cells into the Th17 cells after they have been activated by TGF-b and IL-6. TGFB/TGFBR1 and IL6 are also involved in the differentiation of Th17 cells [90,91]. Some studies indicated IL2/IL2RA complex relates to the differentiation of immune system cells substantially via GM-CSF factor [92]. SMAD4 is phosphorylated by TGFB and is involved in controlling the expression of genes involved in many responses of the immune system and the differentiation of TH17 cells [93]. PRKCQ family plays a crucial role in T-cell activation, it may activate NF-κB and AP-1 [94,95]. Although these differentiation pathways were predicted in our study, it has to be investigated whether they also contribute to the differentiation of all cell lines derived from HSCs.

## 5 Conclusion

The single cell RNA-Seq and ATAC-Seq data could reveal better gene profiles as reported in the differentiated blood cell, myeloid, and lymphoid cell lineages [[96], [97], [98], [99]], however, these data were not enough to analyze the cell cycle phases in GEO database, so that in this study, the transcriptomic data obtained from the gene expression profiling by the array. The high-throughput data analysis suggested the gene profiles that could predict the crucial roles of genes in signaling pathways, however, epigenetic changes, gene mutations, interactions with neighboring cells, and post-translational modifications can affect the functions of signaling pathways. The gene datasets suggested the high- and down-express genes in each of the G0 and G1 phases and the differentiation of HSCs. Furthermore, the gene networks were constructed and enriched using KEGG pathways and GO database so that some signaling pathways were predicted to be highly active in the cycle cell phases. It is also suggested that gene editing techniques such as CRISPR-Cas9, monitoring and adjustment, computational analysis, and in vivo studies should be utilized to consider the exact impact of a gene on various cellular pathways. Moreover, it will be better to enrich the study results by reports of omics such as single-cell RNA sequencing and epigenetic assays, proteomics, and post translational modifications and experimental evaluations that could provide a more comprehensive understanding of the signaling pathways involved in the HSC cell cycle stages.

## Ethics approval and consent to participate

Not applicable.

## Human and animal rights

No animals/humans were used for this review.

## Consent for publication

Not applicable.

## Availability of data and materials

Not applicable.

## Funding

It is supported by Iran University of Medical Sciences (No. 24931).

## CRediT authorship contribution statement

**Mohammad Elahimanesh:** Writing – original draft, Visualization, Validation, Software, Resources, Methodology, Investigation, Formal analysis, Data curation, Conceptualization. **Nafiseh Shokri:** Investigation, Data curation. **Payam Mohammadi:** Methodology, Investigation, Data curation. **Najmeh Parvaz:** Data curation. **Mohammad Najafi:** Writing – review & editing, Writing – original draft, Supervision, Project administration, Conceptualization.

## Declaration of competing interest

The authors declare no conflict of interest, financial or otherwise.

## Figures and Tables

**FigS1:**
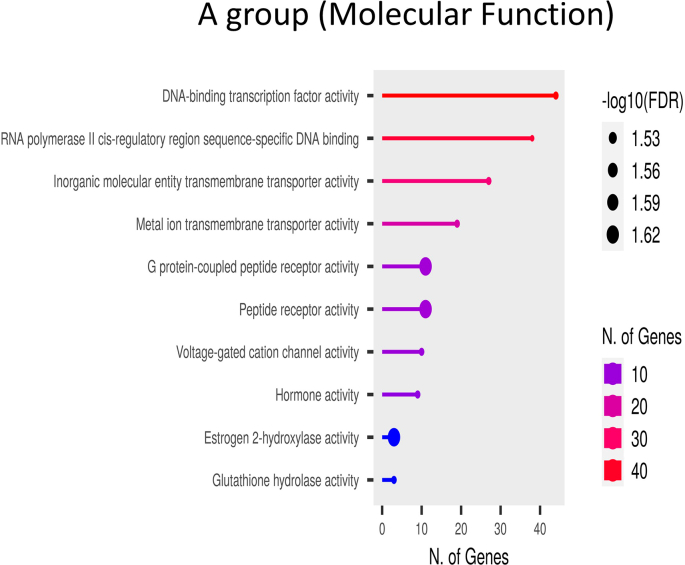


**FigS2:**
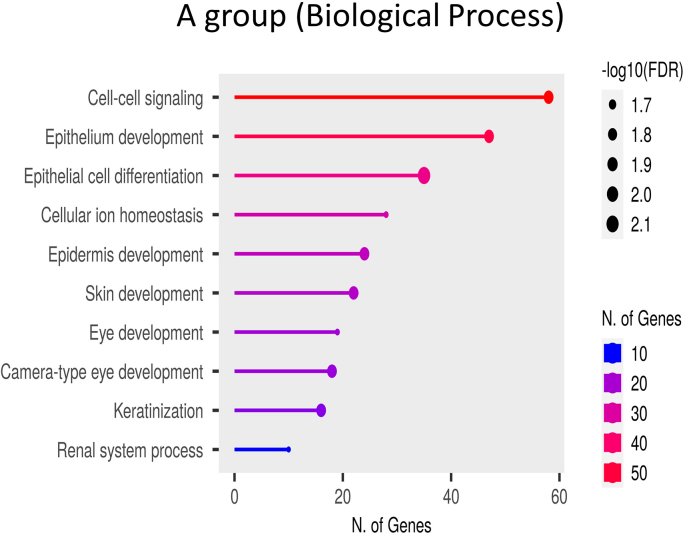


**FigS3:**
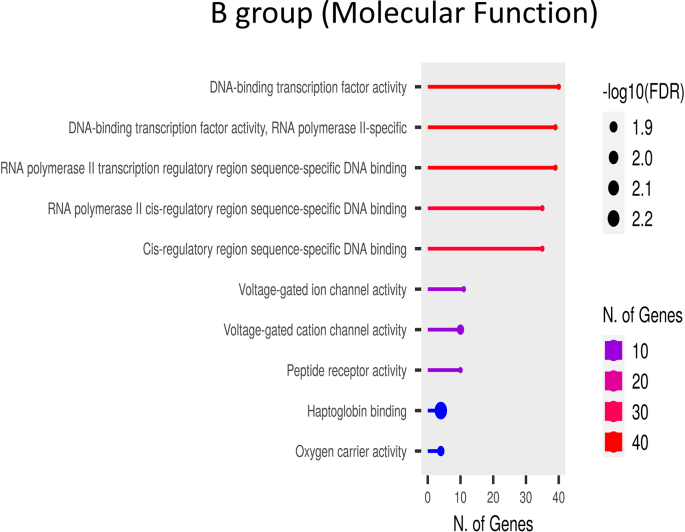


**FigS4:**
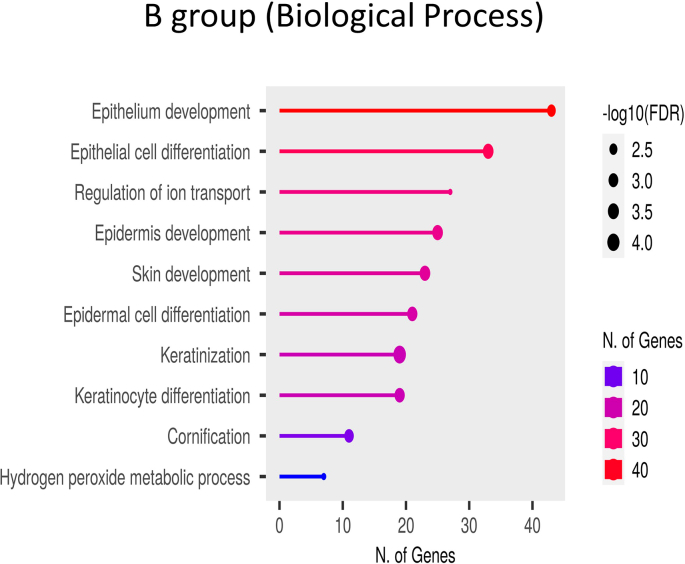


**FigS5:**
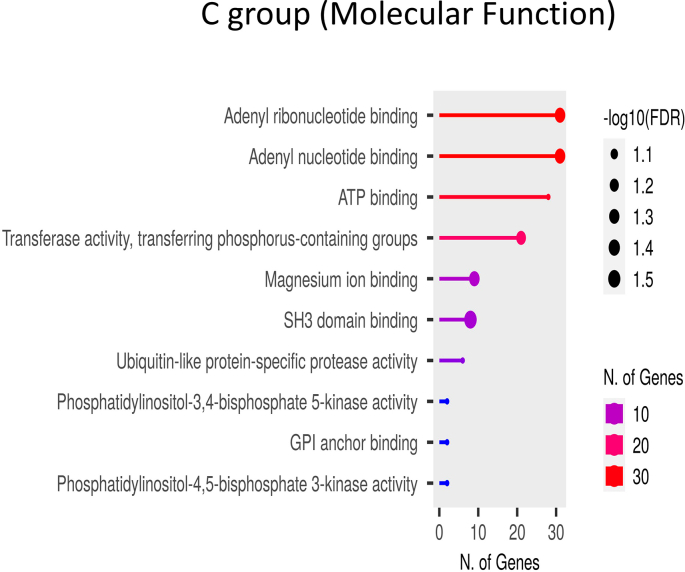


**FigS6:**
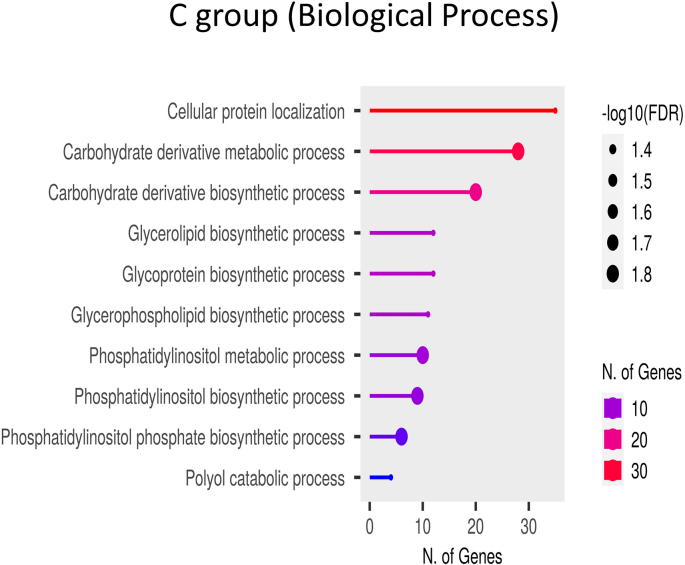


**FigS7:**
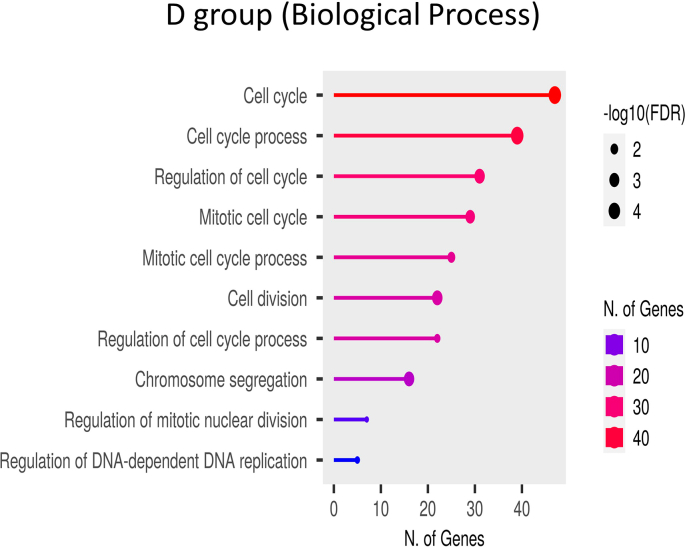


**FigS8:**
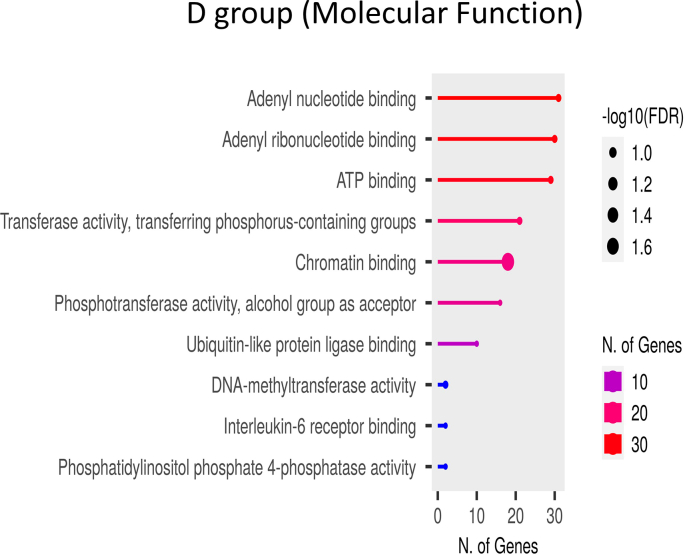


**FigS9:**
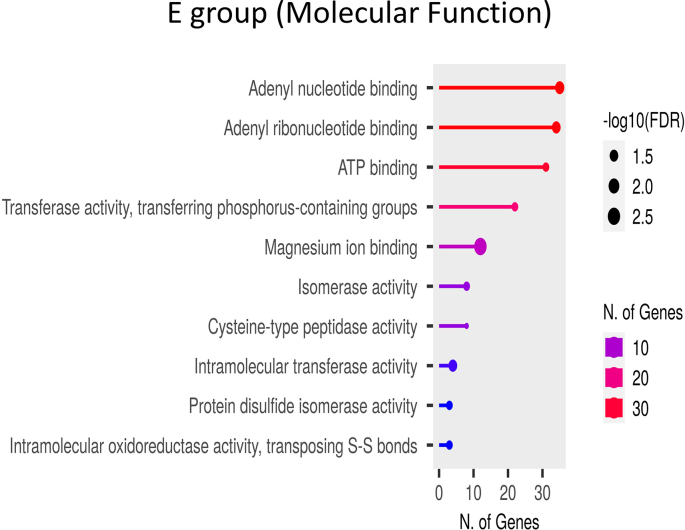


**FigS10:**
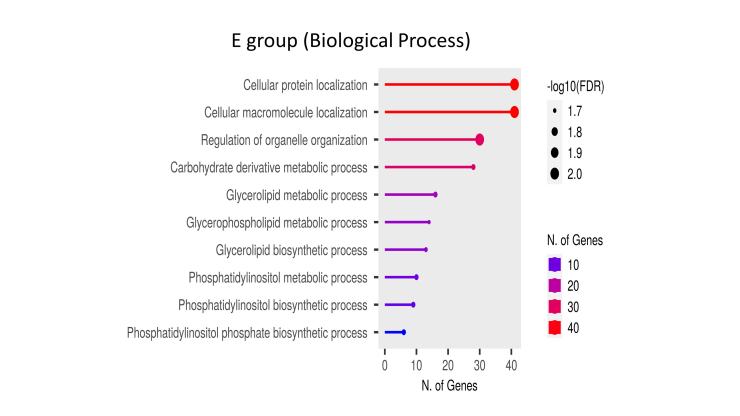


**FigS11:**
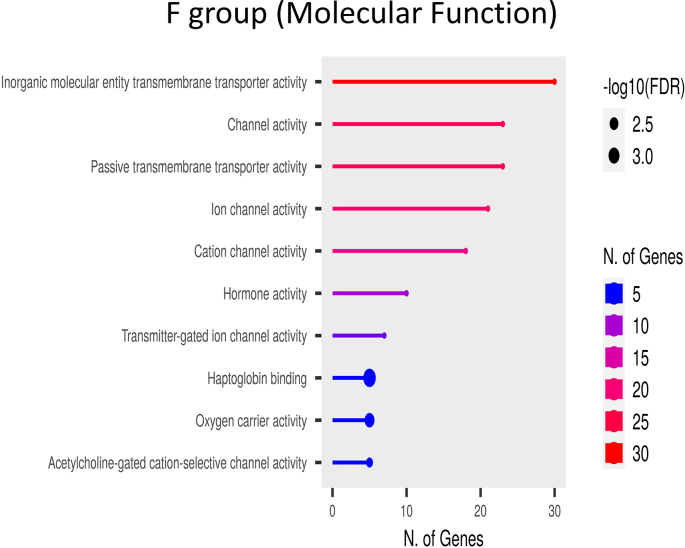


**FigS12:**
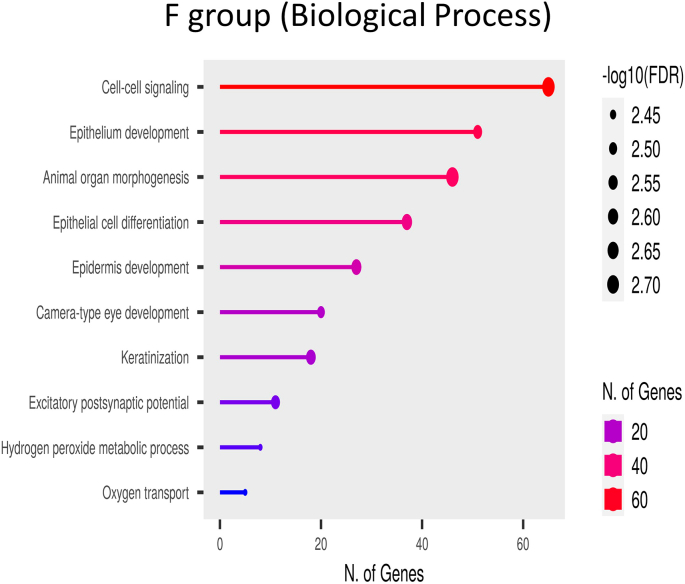


## Data Availability

Data will be made available on request.
